# Systemic endotoxin activity correlates with clot formation: an observational study in patients with early systemic inflammation and sepsis

**DOI:** 10.1186/cc12892

**Published:** 2013-09-11

**Authors:** Alexander Koch, Michael Isaäc Meesters, Bertram Scheller, Christa Boer, Kai Zacharowski

**Affiliations:** 1 Clinic of Anesthesiology, Intensive Care Medicine and Pain Therapy, University Hospital Frankfurt, Theodor-Stern-Kai 7, Frankfurt am Main 60590, Germany; 2 Department of Anesthesiology, VU University Medical Center, De Boelelaan 1117, 1081 HV Amsterdam, The Netherlands

## Abstract

**Introduction:**

Inflammation and coagulation are closely linked, and both can be triggered by endotoxin. Thrombelastometry and impedance aggregometry are of diagnostic and predictive value in critically ill patients. In this observational study we investigated the correlation of endotoxin activity with thrombelasometric and aggregometric variables in patients with systemic inflammation.

**Methods:**

Based on a daily screening on a tertiary academic surgical ICU, patients, as soon as they fulfilled two or more criteria for systemic inflammatory response syndrome (SIRS), were included. In whole blood we performed endotoxin activity (EA) assay, thrombelastometry (ROTEM^®^) and impendance aggregometry (Multiplate^®^).

**Results:**

In total, 49 patients were included with a broad spread of EA levels of (median (minimum to maximum)) 0.27 (0.01 to 0.72), allowing expedient correlative analysis. Clot formation time (CFT) (263 s (60 to 1,438 s)) and clotting time (CT) (1,008 s (53 to 1,481 s)) showed a significant negative correlation with EA level (r = -0.38 (*P *< 0.005) and r = -0.29 (*P *< 0.05)). Positive correlations were found for alpha-angle (50° (17 to 78°), r = 0.40 (*P *< 0.005)) and maximum clot firmness (MCF) (55 mm (5/76), r = 0.27 (*P *< 0.05)). No significant correlations were found between Lysis Index at 60 minutes (LI60) and EA levels. There was no correlation between EA level and aggregometric values, or classical coagulation parameters.

**Conclusions:**

In patients with systemic inflammation, increasing endotoxin concentrations correlate with increased clot formation.

## Introduction

During systemic inflammation, endotoxin (lipopolysaccharide (LPS)), can originate from invading Gram-negative bacteria and/or translocation of endogenous Gram-negative wall fragments. Translocation of endotoxin appears in the context of gastrointestinal hypoperfusion and gut failure, a frequently observed pathophysiology on intensive care units [1]. Subsequently, LPS is released into sterile compartments of the organism and in turn is detected by pattern recognition receptors (PRRs), like toll-like receptor (TLR)4, resulting in initiation of inflammation and coagulation [2,3]. In critically ill patients, concentrations of endotoxins are elevated and measurable with the endotoxin activity (EA) assay. EA levels correlate with Acute Physiology and Chronic Health Evaluation II (APACHE II), the presence of severe sepsis and mortality [4].

The cross-talk between inflammation and coagulation is a central player in the pathophysiology of systemic inflammation and sepsis [5,6]. *In vitro *and *in vivo *models demonstrate the potential of LPS to initiate clotting. Added to whole blood, LPS reduces clotting time (CT) as measured with (rotational) thrombelastometry (TEM) [7,8]. Compared to healthy controls, both postoperative patients and patients with sepsis demonstrate shorter CTs and clot formation times (CFTs) [9]. In septic patients, particularly CFT seems to correlate well with clinical condition and outcome [10].

Furthermore, systemic inflammation alters platelet function, and under certain conditions, sepsis can lead to activation and aggregation of platelets [11-13]. However, more recent data demonstrate reduced platelet aggregation in patients with severe sepsis compared to postoperative patients [14]. The Multiplate^® ^analyzer is a modern type of impedance aggregometer which is designed as a bedside tool (point of care) to assess platelet function [15].

The correlation between EA levels and thrombelastometric or aggregometric measurements during systemic inflammation has not been evaluated yet. The further understanding of the relationship of systemic inflammation and coagulation in patients might be of relevance for the introduction of new therapeutic approaches. For example, endotoxin neutralizing or eliminating concepts could also positively influence coagulatory dysregulations in these patients.

The primary outcome of this prospective observational study was the association of EA level and CFT in patients with early systemic inflammation. Secondary outcomes were the association of EA levels with further thrombelastometric and aggregometric measurements and the influence of infection (that is, sepsis).

## Materials and methods

### Patients

This single center prospective observational study included adult patients with two or more systemic inflammatory response syndrome (SIRS) criteria on a 26-bed tertiary academic surgical ICU. The study was approved by the local ethical review committee (University Hospital Frankfurt, Germany) and carried out in compliance with the principles established in the Helsinki Declaration. Written informed consents were obtained from the patients or legal representatives for patients unable to consent. From April to July 2011, on a daily basis between 06:30 to 07:30 am, all patients were screened for SIRS criteria [16]. Patients fulfilling two or more of the following SIRS criteria were included: (a) core temperature of >38°C or <36°C, (b) heart rate of >90 beats/minute, (c) respiratory rate of >20 breaths/minute or partial pressure of arterial carbon dioxide (PaCO_2_) <32 mmHg (all patients screened were breathing spontaneously), (d) total WBC absolute count >12,000 cells/mm^3 ^or <4,000 cells/mm^3^. Sepsis was diagnosed when additionally, microbiological results revealed an organism grown in blood/sterile sites or infected tissue was detected clinically (for example, pneumonia).

Exclusion criteria: age <18 years, denial of informed consent, chronic liver disease, treatment with anticoagulants other than heparin or low molecular weight heparin (LMWH), history of coagulopathy or thrombophilia.

### Procedures

Demographic variables, routine blood tests, including conventional clotting, clinical and microbiological data were collected. Blood samples were obtained between 08:00 a.m. and 12:00 p.m. (noon) on the day of inclusion. Inclusion bias was avoided by including patients before measurements were performed. Blood samples were drawn from an indwelling arterial line (flush setups were not heparinised) and consisted of one EDTA-, one citrate-tube and one heparinised tube, for EA, thrombelastometric, and aggregometric measurements, respectively. Measurements were performed within one hour. EA levels were determined by Endotoxin Activity Assay (EAA) (EAA™, Spectral Diagnostics Inc., Toronto, ON, Canada), according to the manufacturer's instruction. Rotational thromboelastometry measurements were performed according to the manufacturer's recommendations (ROTEM^®^, Tem International GmbH, München, Germany). A total of 300 μL of citrated blood was pipetted into ROTEM^® ^sample cups and heparinase (to exclude potential effects of heparin) and CaCl_2 _(20 μl heparinase and 0.2 M CaCl_2_, NATEM-test) were added. The NATEM-test was chosen since no activators are added. *In vitro *experiments revealed that endotoxin initiated clotting activation cannot be measured sufficiently by thrombelastometry when performed with additional clotting activators [7]. Furthermore, the NATEM-test has been previously evaluated in patients with systemic inflammation [9,10]. Sample tubes were temperature controlled at 37°C. CT, CFT, maximum clot firmness (MCF), alpha angle and 60-minute lyses index (LI60) were determined. Platelet function was investigated by impedance aggregometry using Multiplate^® ^(Verum Diagnostica GmbH, Munich, Germany) analysis according to the manufacturer´s recommendations. A total of 300 μl heparinised whole blood and 300 μl saline was pipetted into the test cells which were temperature controlled at 37°C. Samples were activated with arachidonic acid (ASPI), adenosine diphosphate (ADP), or thrombin receptor activating peptide 6 (TRAP). Results are given as area under curves of arbitrary aggregation units (A.U.) over time.

### Statistical analysis

Statistical analyses were performed with Prism^® ^5.02 (GraphPad Software, San Diego, CA, USA). The correlation of EA activity units with viscoelastic (ROTEM^®^) and aggregometric (Multiplate^®^) parameters were analyzed by Spearmann's correlation test. Groups were compared using the Mann-Whitney U or Kruskal-Wallis (followed by Dunn's multiple comparison) test for continuous variables and chi-square tests for categorical variables. *P *< 0.05 was regarded as statistically significant.

## Results

A total of 49 patients, all European Caucasians, fulfilled the inclusion criteria during the observation period. Characteristics are given in Table 1. Patients were admitted following trauma, major surgical procedures or for postoperative complications, such as respiratory failure or resuscitation. The median (minimum-maximum) length of stay on ICU before inclusion was 2 (0 to 58) days. In 33 patients infection was present at the time of inclusion. The primary site of infection was the lung, followed by the abdomen. In one patient the blood culture was positive. Other infection sites included muscle tissue or the urinary tract (Table 1). Thrombosis prophylaxis was provided with heparin-infusion in 29 (59.2%) or low molecular weight heparin (LMWH) in 13 (26.5%) patients. Seven (14.3%) patients did not receive thrombosis prophylaxis. The median (minimum-maximum) heparin-infusion rates were 400 (200 to 1,000) units/h. LMWHs included enoxaparin (n = 6) 70 (40 to 120) mg/d, nadroparin-calcium (n = 2) 5,700 (3,800 to 7,600) units/d and dalteparin (n = 5) 10,000 (10,000 to 10,000) units/d. Fourteen (28.6%) patients received antiplatelet therapy (acetylsalicylic acid, 100 mg/d).

**Table 1:** Characterization of patients

Variables	All *n *= 49	SIRS *n *= 16	Sepsis *n *= 33
Age^†^	70 (28/87)	71.5 (53/86)	70 (28/87)
Male^‡^	35 (71.4)	11 (68.8)	24 (72.7)
BMI^†^	27.1 (16.3 to 40.4)	27.5 (20.8 to 27.0)	26.3 (16.3 to 40.4)
Infection^‡^	33 (67.4)	0 (0)	33 (100)
Infection site	See sepsis	NA	Lung: 17
		NA	Abdominal: 8
		NA	Positive blood culture: 1
		NA	Other: 7
Temperature (°C)^†^	36.4 (33.4 to 38.9)	36.5 (35.2 to 38.0)	36.4 (33.4 to 38.9)
Heart rate (bpm)^†^	90 (60 to 140)	90 (70 to 100)	90 (60 to 140)
Respiratory rate (c/min)^†^	15 (10 to 29)	15.5 (11 to 21)	15 (10 to 29)
Leukocytes (×10^9^/L)^†^	14.4 (2.5 to 48.0)	13.6 (7.1 to 28.8)	15.0 (2.5 to 48.0)
Anticoagulation^‡^	Heparin: 29 (59.2)	7 (43.8)	22 (66.7)
	LMWH: 13 (26.5)	6 (37.5)	7 (21.2)
	None: 7 (14.3)	3 (18.8)	4 (12.1)
APACHE II	16 (5 to 29)	16 (10 to 26)	16 (5 to 29)
SAPS II	46 (19 to 90)	43 (24 to 62)	48 (19 to 90)

†, median (minimum-maximum); ‡, n (%); APACHE, Acute Physiology and Chronic Health Evaluation; BMI, body mass index; bpm, beats per minute; LMWH, low molecular weight heparin; SAPS, Simplified Acute Physiology Score; SIRS, systemic inflammatory response syndrome

Patients with SIRS or sepsis did not differ significantly in age, sex, body mass index (BMI), SIRS criteria, type of anticoagulation, Acute Physiology and Chronic Health Evaluation (APACHE) II or Simplified Acute Physiology Score (SAPS) II.

### EA and viscoelastic ROTEM^® ^variables

**Figure 1:**
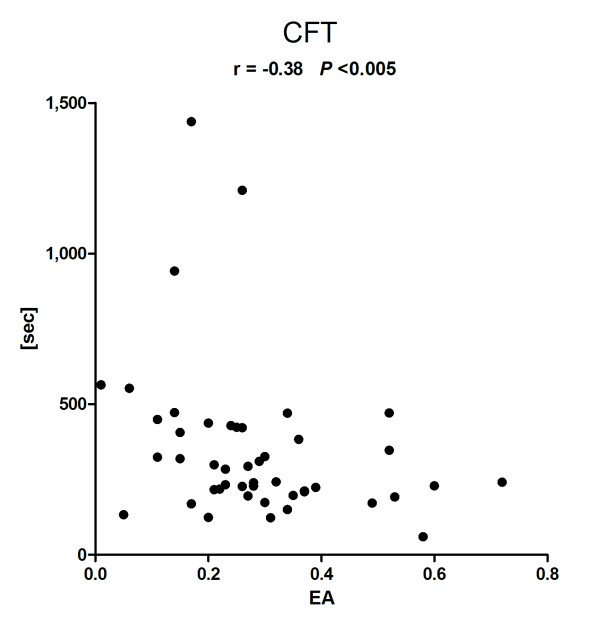
**Endotoxin activity (EA) and NATEM clotting formation time (CFT)**. r, Spearman's correlation coefficient

**Figure 2:**
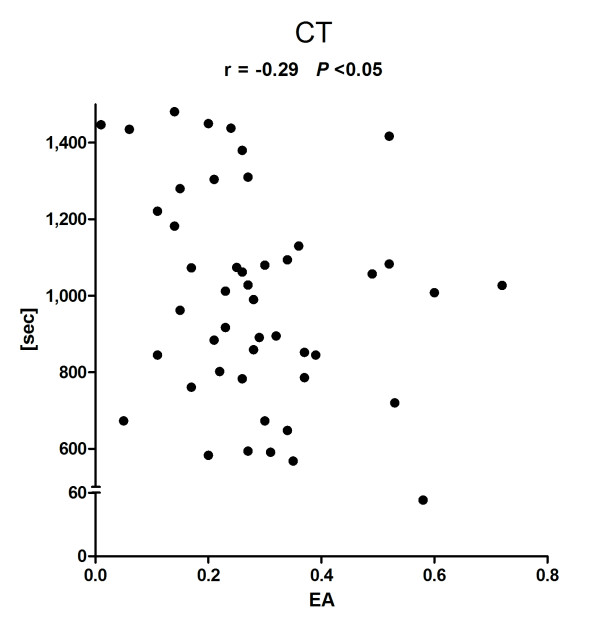
**Endotoxin activity (EA) and NATEM clotting time (CT)**. r, Spearman's correlation coefficient

**Figure 3:**
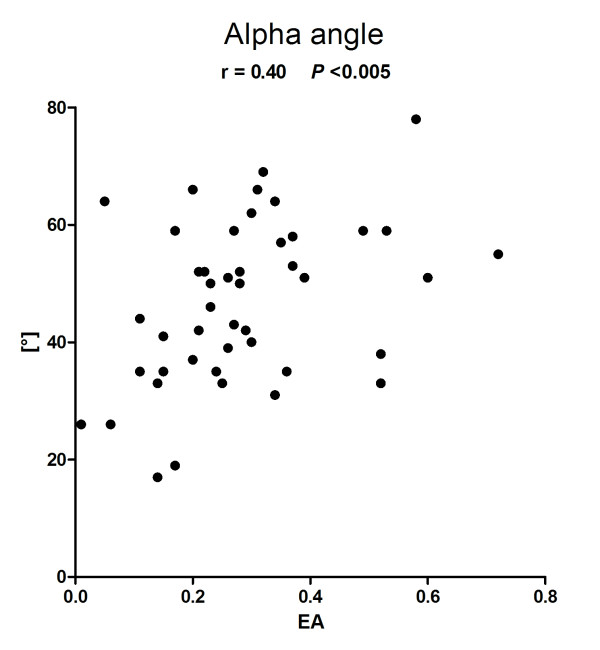
**Endotoxin activity (EA) and NATEM alpha-angle**. r, Spearman's correlation coefficient

EA levels showed a broad distribution with a median (minimum-maximum) of 0.27 (0.01 to 0.72). In two patients, thrombelastometric measurements failed for technical reasons. The median values (minimum-maximum) of the viscoelastic variables were CFT: 263 s (60 to 1,438 s), CT: 1,008 s (53 to 1,481 s), and alpha-angle: 50° (17 to 78°). The primary outcome of this study, that is, the association of EA and CFT, showed a significant negative correlation (r = -0.38, *P *< 0.005) (Figure 1). Furthermore, a negative correlation was found for CT (r = -0.29, *P *< 0.05), a positive correlation was found for alpha-angle (r = 0.40, *P *< 0.005) (Figures 2 and 3). A positive correlation could also be observed for maximum clot firmness (MCF). No correlations were found between EA and Lysis Index at 60 minutes (LI60) (Table 2). Viscoelastic parameters did not differ significantly between patients receiving heparin-infusion, LMWH or no antithrombosis prophylaxis (data not shown).

**Table 2:** Thrombelastometric and aggregometric findings, conventional clotting variables

Variables	Value	Correlation with EA	
		
		r	*P*
CFT (sec)	263 (60 to 1,438)	-0.38	<0.005
CT (sec)	1,008 (53 to 1,481)	-0.29	<0.05
Alpha angle (°)	50 (17 to 78)	0.40	<0.005
MCF (mm)	55 (5 to 76)	0.27	<0.05
LI60 (60%)	98 (90 to 100)	-0.04	NS
ASPI (AUC (AU*min))	697 (53 to 1,481)	0.01	NS
ADP (AUC (AU*min))	518 (18 to 1,176)	0.01	NS
TRAP (AUC (AU*min))	818 (122 to 1,831)	-0.05	NS
Platelets (×10^9^/L)	145 (17 to 523)	0.02	NS
INR	1.39 (1.04 to 3.58)	0.01	NS
APPT (sec)	45 (32 to 112)	0.02	NS
Thrombin time (sec)	16.5 (14.0 to 95.0)	-0.11	NS
Fibrinogen (mg/dL)	440 (60 to 1,106)	-0.10	NS
Antithrombin (%)	62 (27 to 90)	-0.08	NS

ADP, adenosine diphosphate activated aggregometric analysis; aPPT, activated partial thromboplastin time; median (minimum-maximum); ASPI, arachidonic acid activated aggregometric analysis; CFT, clotting formation time; CT, clotting time; INR, international normalized ratio; LI60, 60-minute lyses index; MCF, maximum clot firmness; TRAP, thrombin receptor activating peptide 6 activated aggregometric analysis

### EA and aggregometric Multiplate^® ^variables

No significant relations were found between EA and ASPI-test, ADP-test or TRAP-test (Table 2). Aggregometric parameters and correlations to EA were not influenced significantly by antiplatelet therapy (data not shown).

### EA and conventional clotting variables

No significant correlations were found between EA and platelet count, International Normalized Ratio (INR), activated Partial Thromboplastin Time (aPPT), fibrinogen concentration, thrombin time or antithrombin activity (Table 2).

### SIRS versus sepsis

EA activity, thrombelastometric, aggregometric or conventional clotting parameters did not differ significantly between SIRS and sepsis patients (Table 3).

**Table 3:** EA, thrombelastometric and aggregometric findings, conventional clotting variables in SIRS and sepsis patients

	SIRS	Sepsis	
**Variables**	**Value**	**Value**	***P***

EA	0.27 (0.14 to 0.52)	0.27 (0.01/0.72)	NS
CFT (sec)	263 (150 to 1,438)	268 (60/942)	NS
CT (sec)	906 (648 to 1,380)	1027 (53 to 1,481)	NS
Alpha-angle (°)	50 (24 to 69)	47 (17 to 78)	NS
MCF (mm)	53 (24 to 67)	57 (5 to 76)	NS
LI60 (60%)	98 (90 to 100)	99 (93 to 100)	NS
ASPI (AUC (AU*min))	506 (49 to 1,137)	772 (21 to 1,667)	NS
ADP (AUC (AU*min))	539 (125 to 804)	504 (18 to 1,176)	NS
TRAP (AUC (AU*min))	799 (339 to 1,494)	887 (122 to 1,831)	NS
Platelets (×10^9^/L)	131 (61 to 257)	166 (17 to 523)	NS
INR	1.40 (1.14 to 3.58)	1.37 (1.04 to 2.12)	NS
APPT (sec)	44 (38 to 112)	45 (32 to 79)	NS
Thrombin time (sec)	17 (14 to 95)	16 (14 to 79)	NS
Fibrinogen (mg/dL)	392 (60 to 829)	451 (112 to 1,106)	NS
Antithrombin (%)	57 (31 to 90)	62 (27 to 88)	NS

ADP, adenosine diphosphate activated aggregometric analysis; aPPT, activated partial thromboplastin time; median (min-max); ASPI, arachidonic acid activated aggregometric analysis; CFT, clotting formation time; CT, clotting time; EA, endotoxin activity; INR, international normalized ratio; LI60, 60-minute lyses index; MCF, maximum clot firmness; TRAP, thrombin receptor activating peptide 6 activated aggregometric analysis

## Discussion

In this prospective observational study we investigated the relation between systemic endotoxin levels and clotting dynamics. We found EA levels to negatively correlate with CFT and CT. Positive correlations were found for alpha-angle and MCF, indicating that clot formation was faster and firmer when EA levels were higher. We did not observe any effect of EA levels on LI60, that is, clot lyses. Furthermore, there was no correlation between EA levels and platelet function, as investigated by aggregometric measurements (ASPI-, ADP-, TRAP-test). Also, conventional clotting tests (platelet count, INR, aPPT, thrombin time) or clotting factor measurements (fibrinogen, antithrombin) did not correlate with EA levels. There were no differences in EA levels, thrombelastometric, aggregometric or conventional clotting parameters between SIRS and sepsis patients.

SIRS following major surgery (for example, heart surgery, liver transplantations), burns or major trauma is associated with increased endotoxin blood levels [17-24]. It is thought that endotoxin translocates in the context of gastrointestinal hypoperfusion and gut failure. Thereby, EA in the blood seems to mainly reflect organ dysfunction with regard to the integrity of the gut, which can be observed in patients with SIRS, Gram positive or Gram negative sepsis [25,26]. In patients with systemic inflammation caused by infection, that is, sepsis, EA levels tend to be higher. However, we and others showed that EA levels do not discriminate between SIRS and sepsis [4]. With the intention of measuring a broad range of EA levels, we defined the presence of two SIRS criteria as inclusion criteria, regardless of cause or severity of systemic inflammation. EA levels can be graduated into low (<0.4), intermediate (0.40 to 0.59) and high (≥0.6). The range of EA levels we observed are slightly lower, however, of a comparable broad spread when compared to the MEDIC study [4]. This allowed us to correlate various EA levels with corresponding thrombelastographic and aggregometric parameters. The fact that we observed lower EA levels compared to the MEDIC study could be due to differences in inclusion times. While all patients of the MEDIC study were included within 24 h, the median inclusion time in our study was 2 d after ICU admission. One could speculate that in our population initial stabilisation following, for example, surgery was more advanced, and surgery related endotoxin release was of reduced impact.

In 33 patients, SIRS was associated with infection, resulting in sepsis, severe sepsis or septic shock. Our analysis did not discriminate between the different grades of sepsis, as this would result in small group numbers. Furthermore, we aimed to investigate the mere effect of EA on thrombelastographic and aggregometric parameters, rather than trying to detect differences between groups to which patient allocation often is difficult.

TLR activation results in the intracellular activation of NF-κB and the subsequent release of various cytokines, nitric oxide, several other mediators and tissue factor (TF) [5,27]. Circulating TF is released primarily by activated monocytes, granulocytes and macrophages. Various conditions, including SIRS and sepsis, are related to increased TF levels [28,29]. The central role of TF in systemic inflammation could be demonstrated by blocking TF in *Escherichia coli-*induced septic shock, which increased survival in an animal model [30]. In human volunteers, the injection of LPS leads to a fast increase of TF mRNA in monocytes [31]. TF activates the TF/FVIIa pathway, activating FIX and FX, resulting in thrombin formation [32]. On the other hand, anticoagulatory mediators like antithrombin, protein C and S levels are decreased during systemic inflammation, supporting the procoagulatory state of the clotting system [33,34].

In *in vitro and *animal experiments as well as in healthy subjects, clotting activation by endotoxin could be observed [7,35,36]. In healthy volunteers, low dose endotoxin, compared to placebo, reduces CT, but does not affect CFT or MCF [8]. Thrombelastometric observations performed with NATEM-tests in patients with severe sepsis demonstrate that compared to healthy controls, MCF and alpha-angle were increased, that is, clot formation was firmer [37]. Also performing the NATEM-test, Adamzik *et al*. observed a shorter CT and CFT, and a higher alpha-angle in septic patients compared to healthy probands [9]. As severe sepsis is associated with high EA levels, these results would be in line with our findings. However, some further, earlier studies with ROTEM^® ^in patients with severe sepsis or septic shock are difficult to interpret and probably not comparable with our findings [12,38]. This is because clotting activators were used (INTEM, EXTEM and so on). ROTEM^® ^analysis working with clotting activators, such as the INTEM- or the EXTEM-test, might not be sensitive enough to detect clotting abnormalities caused by systemic inflammation/infection. For example, the EXTEM-test uses TF as an activator. As TF is thought to be a central player during inflammatory induced clotting, using it as an activator might override the effects we aimed to investigate. In our study, we omitted activators by using the NATEM-test with heparinase only. Furthermore, the inclusion time in previous trials often was not focused on early systemic inflammation, but on established sepsis. With the inclusion criteria severe sepsis or septic shock, the inclusion time tends to be later compared with the inclusion criteria SIRS, as microbiology results often are essential for the diagnosis severe sepsis and septic shock.

Recently, it was demonstrated that impedance aggregometry has diagnostic and prognostic value in patients with severe sepsis. Compared to post-surgical patients, patients with severe sepsis showed reduced platelet aggregation in the collagen-test, ADP-test, AA-test and TRAP-test [14]. Our results did not show differences in aggregometric variables between SIRS and sepsis patients. This might be explained by the fact that in SIRS patients, systemic inflammation can be more severe than in post-surgical patients. Furthermore, the sepsis group in our study also included patients with sepsis without organ dysfunction, that is, a less severe form of sepsis compared to severe sepsis. Therefore, in our patient groups (SIRS vs. sepsis) clinically and inflammatory conditions were more contiguous, which might explain the absence of differences in our results. Another study, comparing post-surgical with septic shock patients did not, with the exception of ADP, find differences in aggregometric variables (ASPI, COL, TRAP-test) [12]. Again, investigated patient groups (post-surgical vs. septic shock) differed from ours.

The absence of a correlation between EA levels and conventional clotting parameters, as well as the fact that the groups SIRS and sepsis did not differ in this regard, is in line with previous observations. In septic patients, platelet counts tend to be lower when compared to healthy individuals, but not compared to post surgical or SIRS patients [12,39]. Perioperative systemic endotoxin concentrations do not correlate with platelet counts or aPPT [40,41]. Prothrombin time (PT) (in our study represented by INR) does not differ between controls, patients with sepsis or severe sepsis. Only in patients with septic shock, a prolongation can be observed [42]. Therefore, our results support the insight that global clotting tests are not sensitive enough to detect clotting derangements caused by systemic inflammation, apart from extremes, such as septic shock.

This study focused on early SIRS and sepsis states. With the progress of SIRS or sepsis, EA level and coagulation courses over time become extremely complex by the influence of various unbalanced pro- and anti-inflammatory/pro- and anti-coagulatory mechanisms. This is in line with our observations, which initially included analysis for five consecutive days. With EA levels and coagulatory variables following indefinable courses, we focused on early stages of SIRS and sepsis. Still, the correlations we found are not very intense. This might reflect the above mentioned fast and diverse dynamic of inflammatory and coagulatory processes, whose exact onset cannot precisely be captured by a screening resolution of 24 hours. Potentially, higher numbers of patients would have adjusted this synchronisation deficiency. Furthermore, medical histories and clinical conditions of the patients were very heterogeneous, which should be considered when interpreting the levels of correlation coefficients.

## Conclusion

The impact of endogenously released endotoxin on clotting has not been evaluated before. Here we demonstrate a correlation between EA levels and viscoelastic parameters, suggesting an *in vivo *link between endogenous endotoxin concentrations and coagulation. With increasing EA levels clot formation becomes faster and firmer in patients with early systemic inflammation, independent of cause (non-infectious or infectious) or severity. With the development of endotoxin neutralizing therapeutic options, the further understanding of the link between systemic inflammation and coagulation might be of relevance [25].

## Key messages

• In patients with early systemic inflammation, increasing endotoxin concentration correlates with faster and firmer clot formation.

• Neither platelet counts or function nor conventional clotting parameters were influenced by endotoxin concentration.

• Patients with SIRS did not differ from patients with sepsis in endotoxin concentrations, clot formation, platelet counts or function, or conventional clotting parameters.

## Abbreviations

ADP, adenosine diphosphate activated aggregometric analysis; APACHE, Acute Physiology and Chronic Health Evaluation; aPPT, activated partial thromboplastin time; ASPI, Arachidonic Acid Activated Aggregometric Analysis; BMI, body mass index; bpm, beats per minute; CFT, clot formation time; CT, clotting time; EA, endotoxin activity; EAA, Endotoxin Activity Assay; EDTA, ethylenediaminetetraacetic acid; INR, international normalized ratio; LI60, 60-minute lyses index; LMWH, low molecular weight heparin; LPS, lipopolysaccharide; MCF, maximum clot firmness; PRRs, pattern recognition receptors; PT, prothrombin time; SAPS, simplified acute physiology score; SIRS, systemic inflammatory response syndrome; TF, tissue factor; TEM, thrombelastometry; TLR, toll-like receptor; TRAP, thrombin receptor activating peptide 6 activated aggregometric analysis

## Competing interests

The authors declare that they have no competing interests.

## Authors' contributions

AK designed the study, screened and enrolled patients, analyzed and interpreted the data, and wrote the manuscript. MIM screened patients, performed the bedside measurements, collected patient data, analyzed and interpreted the data, and revised the manuscript for important intellectual content. BS assisted in processing and analyzing the data, and revised the manuscript for important intellectual content. CB obtained funding and revised the manuscript for important intellectual content. KZ conceived of the study, obtained funding, participated in its design and coordination, headed the project, and revised the manuscript for important intellectual content. All authors read and approved the final manuscript.
